# CYP450 Epoxygenase Metabolites, Epoxyeicosatrienoic Acids, as Novel Anti-Inflammatory Mediators

**DOI:** 10.3390/molecules27123873

**Published:** 2022-06-16

**Authors:** Zeqi Shi, Zuowen He, Dao Wen Wang

**Affiliations:** 1 Hubei Key Laboratory of Genetics and Molecular Mechanism of Cardiological Disorders, Wuhan 430030, China; zeqishi@hust.edu.cn; 2 Division of Cardiology, Department of Internal Medicine, Tongji Hospital, Tongji Medical College, Huazhong University of Science and Technology, Wuhan 430030, China

**Keywords:** arachidonic acid, cytochrome P450 epoxygenase, epoxyeicosatrienoic acids (EETs), soluble epoxide hydrolase (sEH), inflammation

## Abstract

Inflammation plays a crucial role in the initiation and development of a wide range of systemic illnesses. Epoxyeicosatrienoic acids (EETs) are derived from arachidonic acid (AA) metabolized by CYP450 epoxygenase (CYP450) and are subsequently hydrolyzed by soluble epoxide hydrolase (sEH) to dihydroxyeicosatrienoic acids (DHETs), which are merely biologically active. EETs possess a wide range of established protective effects on many systems of which anti-inflammatory actions have gained great interest. EETs attenuate vascular inflammation and remodeling by inhibiting activation of endothelial cells and reducing cross-talk between inflammatory cells and blood vessels. EETs also process direct and indirect anti-inflammatory properties in the myocardium and therefore alleviate inflammatory cardiomyopathy and cardiac remodeling. Moreover, emerging studies show the substantial roles of EETs in relieving inflammation under other pathophysiological environments, such as diabetes, sepsis, lung injuries, neurodegenerative disease, hepatic diseases, kidney injury, and arthritis. Furthermore, pharmacological manipulations of the AA-CYP450-EETs-sEH pathway have demonstrated a contribution to the alleviation of numerous inflammatory diseases, which highlight a therapeutic potential of drugs targeting this pathway. This review summarizes the progress of AA-CYP450-EETs-sEH pathway in regulation of inflammation under different pathological conditions and discusses the existing challenges and future direction of this research field.

## 1. Introduction

Inflammation is a complex and highly orchestrated process that plays a crucial role in the initiation and development of a wide range of systemic illnesses [1,2]. The majority of the enzymes, eicosanoid metabolites, and receptors of the arachidonic acid pathways are critical therapeutic targets, especially for inflammation [3]. The first pathway to be targeted in arachidonic acid metabolism was the cyclooxygenase (COX) pathway, which produces prostaglandins. Drugs targeting this pathway, including aspirin and non-steroidal anti-inflammatory drugs (NSAIDs), are effective at treating pain and inflammation [4,5]. The production of leukotrienes via lipoxygenase (LOX) was the second pathway to be targeted therapeutically. Antagonists for leukotriene receptor and Arachidonate 5 lipoxygenase (ALOX5) have been developed for the treatment of seasonal allergies and asthma [6,7]. These two eicosanoid pathways are becoming increasingly important as novel metabolites and eicosanoid receptors are discovered and their properties in diseases are well defined.

The cytochrome P450 pathway, which is considered as the third eicosanoid pathway, was first described in 1980 [8]. The CYP450 pathway produces four regioisomers of epoxyeicosatrienoic acids, 5,6-EET, 8,9-EET, 11,12-EET, and 14,15-EET, which are hydrolyzed by the sEH to DHETs with very low biological activity [3]. In contrast to the previous two pathways, knowledge of the CYP450 pathway is rather limited and has not yet been exploited therapeutically [9,10,11,12]. EETs have been identified as autocrine and paracrine signaling lipid mediators, which have vasodilative, anti-hypertensive, cardio-protective, renal-protective, pro-angiogenic, and metabolic-regulatory effects [11]. Beyond the well-established protective properties of EETs, a growing body of evidence has shown that inflammatory states decrease the expression and activity of CYP450 enzyme [13], suggesting EETs are potential endogenous anti-inflammatory mediators. Meanwhile, EETs attenuated endothelial activation, leukocyte adhesion, platelet aggregation, and adventitial fibroblast activation, and therefore inhibited vascular inflammation and remodeling [14,15]. EETs also process direct and indirect anti-inflammatory properties in myocardium and therefore prevent the development of inflammatory cardiomyopathy and cardiac remodeling [16,17]. Beyond the well-established role of EETs in cardiovascular system, there are emerging studies showing the anti-inflammatory roles of EETs under numerous diseased states, such as diabetes, sepsis, lung injuries, neurodegenerative disease, hepatic diseases, kidney injury, and arthritis [3,18,19,20]. Given the significant anti-inflammatory effects of EETs, pharmacological, and genetic manipulations of the AA-CYP450-EETs-sEH pathway could be a promising therapeutic strategy for inflammatory disease. This review article focuses on the progress of AA-CYP450-EETs-sEH pathway in inflammatory pathophysiology, as well as the existing challenges and future direction of this research field.

## 2. Generation and Metabolism of EETs

**Figure 1:**
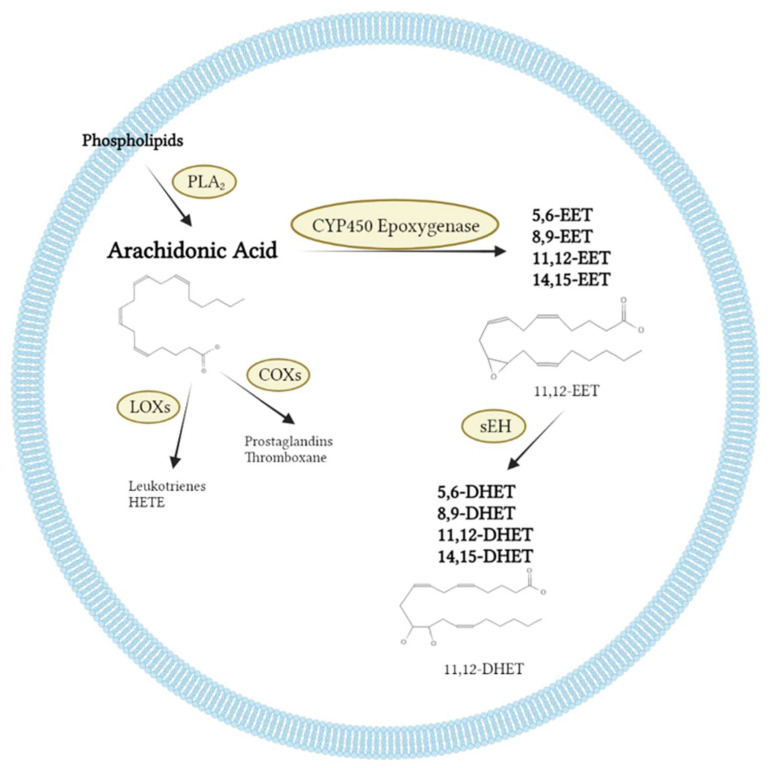
Schematic figure of arachidonic acid metabolism. Membrane phospholipids are converted to AA by PLA_2_. The COXs metabolize AA to PGs and TXAs. The LOXs metabolize AA to LTs and HETEs. The CYP450 (including the 2J and 2C families) metabolize AA to four regioisomers of EETs, 5,6-EET, 8,9-EET, 11,12-EET and 14,15-EET, respectively; the EETs are hydrolyzed by the sEH to DHETs with very low biological activity. (AA, arachidonic acids; EETs, epoxyeicosatrienoic acids; COXs, cyclooxygenases; LOXs, lipoxygenases; CYP450, CYP450epoxygenases; PGs, prostaglandins; TXA2, thromboxane A2; LTs, leukotrienes; sEH, soluble epoxide hydrolase; HETE, hydroxyeicosatetraenoic acids; DHETs, dihydroxyeicosatrienoic acids).

Arachidonic acid is one of the most abundant omega-6 polyunsaturated fatty acids in the body and is mainly produced by the metabolism of phospholipid membranes via phospholipase A2 (PLA2) [10,21,22]. AA can be metabolized by COX and LOX to produce prostaglandins and leukotrienes, respectively [23]. The two metabolic pathways not only process a large spectrum of physiological and pathophysiological effects, but also have been transformed as therapeutic targets for a variety of diseases, for example the clinical application of prostaglandins, aspirin, and leukotriene receptor antagonists [22]. The CYP450 pathway was firstly described as the third AA metabolizing pathway in 1980 [8]. The CYP450 family of enzymes contains many subclasses of which the ω-hydroxylase and epoxygenase activity are the most important for the metabolism of AA [3]. ω-hydroxylase (mainly CYP4A and CYP4F) catalyze the hydroxylation of AA to generate 19-hydroxyeicosatetraenoic acid (19-HETE) and 20-hydroxyeicosatetraenoic acid (20-HETE) [24]. CYP450 epoxygenases, another important branch of the CYP450 enzymes, catalyze the epoxidation of AA to generate EETs that include four regioisomers 5,6-, 8,9-, 11,12-, and 14,15-EET [10] (Figure 1).

Among cytochromeP450 epoxygenase, CYP2 family is the primary enzyme to generate EETs, mainly including the 2C and 2J classes [25]. The tissues expressing CYP2C and CYP2J mainly include the heart, vessel, kidney, lung, and pancreas. The cell type expressing CYP2C and CYP2J mainly include epithelial cells, endothelial cells, smooth muscle cells, cardiomyocytes, autonomic ganglion cells, and islet cells [26,27,28,29,30,31,32,33]. Specifically, CYP2C8 is expressed mainly in the endothelium, CYP2C9 is expressed mainly in the kidney, and CYP2J2 is expressed mainly in the endothelium and cardiomyocytes [14]. sEH is the product of the EPHX2 gene, and it is the metabolic enzymes of EETs that can hydrolyze EETs to the DHETs, which are not biologically active compared to the EETs (Figure 1). The recent studies have shown that sEH is widely expressed in human and animal tissues. Genetic deletion or pharmacological inhibition of sEH increase the level of EETs by inhibiting the effect of sEHs that hydrolyze the epoxide bonds in EETs [34].

## 3. The Anti-Inflammatory Effects of EETs in Heart Disease

### 3.1. EETs and Inflammatory Cardiomyopathy

Inflammatory cardiomyopathy is defined as inflammation of the heart muscle associated with impaired function of the myocardium that has infectious and noninfectious causes [35]. The key pathophysiological mechanisms of inflammatory cardiomyopathy are considered as serious immune and inflammatory responses, which downregulated the expression of CYP450 and upregulated the expression of sEH, leading to decreased level of EETs [13]. Moreover, we found that glucocorticoids alleviated viral myocarditis, hemodynamic disorders, and cardiac dysfunction, and these protective effects were highly correlated with elevated levels of cardiac EETs [36], suggesting a potential anti-inflammatory effect of EETs in inflammatory cardiomyopathy. In line with these findings, EETs alleviated lipopolysaccharide (LPS)-induced myocardial injury and cardiac dysfunction and promoted the transformation of M1-type macrophages into M2-type macrophages. The specific molecular mechanism was that EET inhibited nuclear factor-κB (NF-κB) activity through PPARα/γ and HO-1 signaling pathway [16]. These results suggest that increasing the activity or expression of CYP450 represents a novel approach to alleviate inflammatory cardiomyopathy caused by infectious factors, such as viral myocarditis and septic cardiomyopathy.

Beyond the potent protective effects of EETs in infectious inflammatory cardiomyopathy, EETs also prevented the non-infectious inflammatory cardiomyopathy. EETs derived from CYP2J2 overexpression prevented TNF-α induced cardiac inflammatory injury and dysfunction by inhibiting apoptosis, reducing inflammation, and enhancing PPARγ expression [37]. Moreover, treatment of doxorubicin induced cardiac dysfunction, cardiomyocyte injury, and apoptosis with marked increased level of CK, cTnI, and stress protein. These toxic effects were closely associated with the inflammatory response and the consequent alteration of the expression of several CYP450 and sEH enzymes, suggesting a role of EETs in alleviating toxic inflammatory response. Meanwhile, CYP2J2 overexpression in myocardium significantly inhibited the above toxic injury induced by doxorubicin. These protective effects of CYP2J2 are related to the increase of EETs level and the inhibition of mitochondrial damage [38]. In alcoholic cardiomyopathy model, CYP2J2-derived EETs attenuated ethanol-induced myocardial dysfunction through inducing autophagy and reducing apoptosis via regulating AMP-activated protein kinase (AMPK)/mTOR signaling pathway [39]. Additionally, sEH deficiency with increased level of EETs played an important role in attenuating lipotoxic cardiomyopathy and dysfunction by regulation of autophagy via AMPK-mTORC signaling pathway [40].

### 3.2. EETs and Cardiac Remodeling

Cardiac remodeling is described as changes in the size, shape, and function of the heart resulting from cardiac overload and injury [41]. Inflammation plays an important pathophysiological role in cardiac remodeling as evidenced by activation of immune cells, production of inflammatory cytokines, tissue injury, and organ damage [42]. The recruited immune cells and the damaged resident cells produce pro-inflammatory mediators, pro-fibrotic mediators, and reactive oxygen species (ROS) that initiate remodeling response and therefore lead to cardiac dysfunction [43,44]. Interestingly, various studies indicated that AA-CYP450-EETs-sEH alleviated cardiac remodeling under different pathophysiological states. Cardiomyocyte-specific expression of CYP2J2 reduced expression of cardiac remodeling proteins (TIMP1, TGF-b1, p-smad3, a-SMA, and collagen I) and subsequently inhibited cardiac dysfunction. These protective effects of CYP2J2 overexpression were mediated by attenuating oxidative stress-mediated NF-kBp65 nuclear translocation via PPAR-γ activation, which is considered as a classical anti-inflammatory pathway of EETs [44]. Meanwhile, CYP2J2 and EETs enhanced Akt1 nuclear translocation through interaction with AMPKα2β2γ1 and subsequently attenuated cardiac hypertrophy [45]. In addition to the protective effects of cardiomyocytes, we found that EETs inhibited the fibrotic response of fibroblasts, including the inhibition of fibroblast proliferation, migration, trans-differentiation, and collagen secretion. This anti-fibrotic effect was achieved by inhibiting G_α12/13_ and its downstream RhoA/ROCK pathway via NO/cGMP signaling [17]. Additionally, EETs derived from CYPJ2 overexpression suppressed the transmission of inflammation from cardiomyocytes to macrophages and therefore prevent cardiac fibrosis and cardiac dysfunction. Consistent with CYP450 overexpression, both sEH gene deletion and inhibition attenuated angiotensin Ⅱ (Ang II)-induced cardiac hypertrophy [46,47]. However, cardiac fibrosis and dysfunction was reduced by sEH inhibition rather than gene deletion. The discrepant phenomena are attributed to the fact that global EPHX2 deficiency leads to a total defect in the sEH metabolic pathway with higher ratio of EETs to DHETs, and consequently caused an adaptive shift in AA metabolism to other proinflammatory pathways [47].

EETs also exhibited anti-remodeling effects in other pathological conditions. EETs derived from overexpression of CYP2J2 by adeno-associated virus (AAVs) significantly reduced ISO-induced cardiac remodeling and dysfunction by inhibiting internal plasma reticulum stress via regulating calcium homeostasis and the expression and activity of SERCA2 [48]. Similarly, sEH inhibitor TPUS inhibited ISO-induced hypertrophy of H9c2 cardiomyocytes [49]. In the context of TAC induced cardiac remodeling, the cardiomyocytes’ specific expression of CYP2J2 not only inhibited cardiac hypertrophy, but also significantly alleviated the electrical remodeling [50].

**Figure 2:**
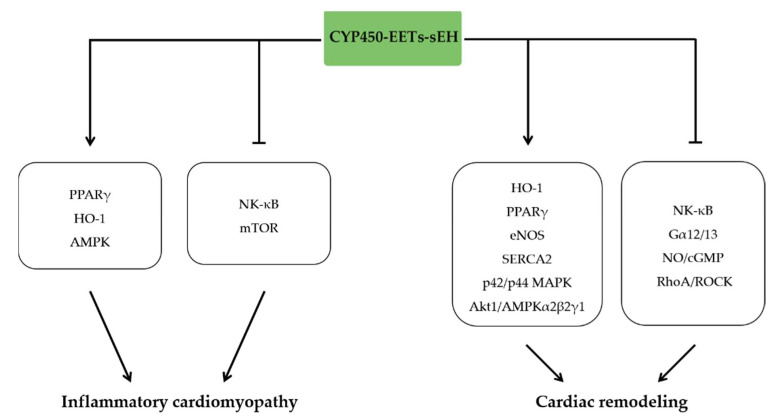
The anti-inflammatory effects of EETs in heart disease. EETs protect against inflammatory cardiomyopathy by activating PPARγ, HO-1, and AMPK signaling pathways and inhibiting NF-κB and mTOR signaling pathways. EETs also attenuate cardiac remodeling by enhancing HO-1, PPARγ, SERCA2, and MAPK and inhibiting NF-κB, Gα12/13 signaling pathways.

Coronary heart disease (CHD) is a common heart disease and one of the leading causes of cardiac remodeling. A large number of evidences demonstrated that polymorphisms in the CYP2J2 gene affect the risk and incidence of coronary heart disease in specific populations [51], and the plasma level of EETs is significantly elevated in patients with coronary artery disease without any change in DHETs levels [52]. Moreover, EET synthesis was increased in stenosed coronary artery [53], and inhibition of sEH resulted in potent vasodilatation of the coronary artery. These results suggest that elevation of EETs levels potential benefits to CHD patients. Myocardial infarction, the most serious type of coronary artery diseases, is accompanied with the inflammatory response in all of the pathophysiological stages. Cardiomyocyte specific expression of CYP2J2 improved cardiac function by increasing the concentration of circulating EETs, and therefore promoted angiogenesis via the Jagged1/Notch1 signaling pathway in myocardial infarction [54]. In post-infarcted stage, administration of an EET analog NUDSA attenuated cardiac fibrosis and inflammation, which were associated with increased neovascularization and increased adiponectin and p-endothelial nitric oxide synthase (eNOS) levels with a concomitant decrease in Bach1 and oxidative stress by HO-1 upregulation [55]. Cardiac ischemic-reperfusion is often accompanied with MI after the reperfusion therapy. EETs derived from CYP2J2 overexpression protect against ischemia-reperfusion in the heart by activation of mito K_ATP_ and p42/p44 mitogen-activated protein kinases (MAPK) [56]. Similarly, 11,12-EET and 14,15-EET significantly reduced myocardial infarct size in ischemic-reperfusion rat model by activation of both the sarc K_ATP_ and mito K_ATP_ channel [57] (Figure 2).

## 4. The Anti-Inflammatory Effects of EETs in Vascular Disease

### 4.1. EETs and Endothelial Protection

Endothelial dysfunction, leukocyte-endothelial adhesion, and subsequent leukocyte transmigration across the endothelium are crucial in the vascular inflammation and involved in the pathogenesis of various vascular diseases, including atherosclerosis, vascular remodeling, and hypertension [58]. Previous research demonstrated that 14,15-EET treatment enhanced U937 cell (a human monocytic cell line obtained from histiocytic lymphoma) adhesion to endothelial cells [59]. Similar effects were observed that overexpression of CYP2C9 enhanced NF-κB activity and the expression of vascular cell adhesion molecule-1 (VCAM-1) [60]. These effects indicate that 14,15-EET may play a substantial role in vascular inflammation. Nevertheless, subsequent studies demonstrated that EETs derived from CYP epoxygenase, especially 11,12-EET, alleviated endothelial dysfunction and leukocyte-endothelial interactions in various model [27,60,61,62,63].

Various studies indicated that the activation of NF-κB induces transcriptional upregulation of endothelial cytokine, chemokine, and cellular adhesion molecule (CAM) expression and drives the subsequent leukocytes adhesion to the endothelium, which is crucial for inflammatory response of blood vessels [64]. Previous data demonstrated that EETs derived from CYP450 epoxygenase played a potent anti-inflammatory effect in the blood vessels by inhibiting NF-κB activation [27]. Moreover, 11,12-EET significantly downregulated expression of VCAM-1, E-selectin, and intercellular adhesion molecule-1 (ICAM-1) in cultured human umbilical vein endothelial cells (HUVECs) treated with TNF-α [27]. Interestingly, these effects were independent of the EDHF properties, as the effects of 11,12-EET on TNF-α-induced VCAM-1 expression was not affected by BK_Ca2+_ channel inhibitor charybdotoxin or iberiotoxin. Furthermore, 11,12-EET has the largest potency to downregulate VCAM-1 expression among the four EET regioisomers, followed by 8,9-EET and 5,6-EET. However, 14,15-EET administration yielded no anti-inflammatory effect. Similarly, intra-arterial infusion of 11,12-EET reduced expression of VCAM-1 in isolated perfused murine carotid arteries after administration of TNF-α intraperitoneally and reduced U937 mononuclear cell adhesion to carotid artery wall induced by TNF-α [27]. Mechanistically, 11,12-EET inhibited TNF-α-stimulated IκB kinase (IKK) activation, inhibitor κB-α (IκB-α) degradation, and subsequent RelA translocation into endothelial cell nuclei, and therefore inhibited cytokine-induces endothelial activation and leukocyte adhesion [27].

Subsequent studies also demonstrated that exogenous administration of 11,12-EET attenuated TNF-α-induced NF-κB activation in HUVECs [60], and 11,12-EET, as well as other isomers, inhibited VCAM-1 upregulation in human saphenous vein endothelial cells (HSVEC) treated with TNF-α [61]. Moreover, the inclusion of 5,6-, 8,9-, 11,12-, or 14,15-EET, together with a specific sEH inhibitor AUDA in cultured media, inhibited TNF-α-induced IκB degradation. However, the protective potency of EETs was significantly reduced in the absence of AUDA, which indicated that the EETs are rapidly degraded by sEH in endothelial cells [14]. A subsequent study indicated that EETs are PPARγ ligands and therefore exert the anti-inflammatory effect by inhibiting IκB degradation [63]. These results suggest that the anti-inflammatory effect of EETs in blood vessels is mediated, at least in part, by inhibiting cytokine-mediated NF-κB activation via PPARγ [63].

### 4.2. The Role of EETs in Vascular Remodeling

Vascular remodeling is an active process of altering structure regulated by the dynamic interaction of locally generated growth factors, inflammatory cytokines, vasoactive substances, and hemodynamic stimuli [65]. Atherosclerosis is a common type of vascular remodeling characterized by inflammation and lipid deposition within the arterial wall, leading to plaque formation [66]. Study showed that sEH inhibition reduced circulating level of LDL by 30%, increased level of HDL by 43%, and elevated the HDL to LDL ratio by 96% [67]. Overexpression of CYP2J2 in mice with an elevation of EETs level reduced low-density lipoprotein and elevated high-density lipoprotein levels by activating PPAR-γ receptor [15], which is considered as a classical anti-inflammatory pathway. These effects suggest EETs have a great potential in preventing atherosclerosis and the coronary artery diseases. Consistently, either specific expression of CYP2J2 in ApoE^−/−^ mice or pharmacological inhibition of sEH significantly increased levels of EETs in mice and subsequently delayed the progression of atherosclerosis by decreasing the expression of adhesion molecules and the level of inflammatory cytokines [68,69,70]. EETs derived from endothelial CYP2J2 overexpression or sEH inhibition reduced adhesion factors expression including ICAM-1, VCAM-1, and suppressed macrophage infiltration, and inflammatory factors release [71]. Accumulative evidence revealed that sEH gene deletion alleviated atherosclerosis by reducing macrophages infiltration [72]. Pharmacological inhibition of sEH by TPPU treatment firmly reduced ER stress and alleviated atherosclerosis caused by endothelial Nox4 dysfunction [73], which is considered as a major determinant of atherosclerosis under atherosclerosis-prone conditions [74]. Abdominal aortic calcification is considered as a marker of coexistent atherosclerotic disease. Recent studies indicated that primary aldosteronism (PA) patients exhibited more severe abdominal aortic calcification (AAC), which is accompanied by higher serum levels of 14,15-DHET. On the contrary, decreased 14,15-EET was significantly associated with AAC prevalence in PA patients [75]. Above all, EETs and sEH inhibition decrease inflammation and have vascular protective actions that can combat atherosclerosis.

**Figure 3:**
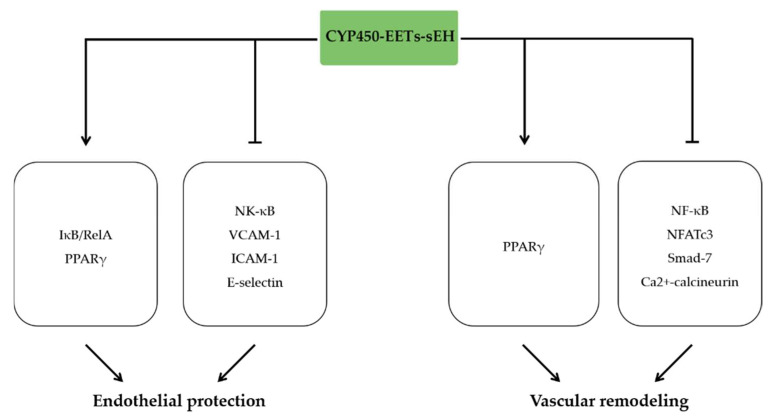
The anti-inflammatory effects of EETs in vascular disease. EETs alleviate endothelial dysfunction by activating PPARγ, IκB activation, RelA translocation, and inhibiting NF-κB, VCAM-1, ICAM-1, E-selectin expression. EETs attenuate vascular remodeling by increasing PPARγ signaling pathway and inhibiting NF-κB, NFATc3, and Smad-7 signaling.

Beyond the well-established role of EETs in alleviation of atherosclerosis characterized as inward remodeling, EETs also potently attenuated outward remodeling. sEH inhibitor TPPU pretreatment with elevation of EETs levels prevented Ang II-induced adventitial remodeling and primary adventitial fibroblasts activation, characterized by differentiation, proliferation, migration, and collagen synthesis via Ca^2+^-calcineurin/NFATc3 signaling pathway in vitro [76]. Consistently, EETs derived from rAAV-CYP2J2 delivery prevented Ang II-induced aortic MMPs expression and activity, elastin degradation, and abdominal aortic aneurysm (AAA) formation and development. Cellular experiments showed that EETs, particularly 11,12-EET, suppress production of inflammatory cytokines in VSMCs and macrophage migration induced by Ang II. Mechanistically, these protective effects were mediated by the EET/PPARγ/NF-κB pathway [15]. Similarly, treatment with an sEH inhibitor attenuated AAA formation and atherosclerosis development. The downregulation of inflammatory mediators and lipid lowering effects may contribute to the observed vascular protective effects [71] (Figure 3).

## 5. The Anti-Inflammatory Effects of EETs in Metabolic Disorder

### 5.1. EETs and Diabetes

Diabetes is a complex metabolic dysfunction affecting the status of glucose in the human body. The majority of people with diabetes fall into two broad pathogenetic categories, type 1 or type 2 diabetes, which are closely associated with metabolic inflammatory response [77]. Previous studies have shown that disorders of glucose metabolism led to activation of neutrophils and macrophages, which cause release of pro-inflammatory cytokines and insulin resistance associated with NLRP3 inflammasome activation [78,79,80].

Adipocyte and adipose tissue dysfunction are essential for pathogenesis of metabolic diseases. EETs are abundant in preadipocytes or mesenchymal stem cells. In contrast, the concentrations of EETs were decreased in mature 3T3-L1 differentiated adipocytes, especially in the early phase [81,82]. The effects suggest that EETs may regulate the function and maturation of adipocytes. Subsequent studies indicated that EETs increased peroxisome proliferation-activated receptor γ coactivator α (PGC-1α) expression, and improved mitochondrial function in the 3T3-L1 preadipocyte maturation [83]. EETs level was significantly suppressed in adipose tissue from diet-induced obesity model [84]. Meanwhile, exogenous treatment of EETs analogs inhibited adipogenesis by suppression of PPARγ, C/EBPα, and SREBP1c mRNA levels in the early phase [84]. Similarly, suppression of HO and EET systems in MSCs was prior to the occurrence of adipocyte dysfunction, which was alleviated by induction of HO-1 and CYP epoxygenase [85]. Beyond the direct regulation of adipocyte, EETs regulated macrophage polarization in adipose tissue by inhibiting cAMP-EPAC signaling pathway and led to metabolic homeostasis and decreased inflammation [86].

Type 1 diabetes (T1DM) is a disease characterized by insufficient insulin production caused by pancreatic β-cell damage [87]. Previous studies indicated that sEH expression is decreased in liver and kidney and is rescued by insulin treatment in type I diabetes mice model [88,89]. However, another research indicated that sEH expression and activity were significantly increased [90]. These studies suggest that EETs-sEH pathway potentially regulates pathophysiology of T1DM, which was confirmed by various models with EET treatment or sEH manipulation. EETs and 8,9-DHET inhibited inflammation by reducing NF-κB activity and nitrite accumulation, and therefore attenuated cytokine-induced apoptosis of pancreatic β-cells [91]. Deletion of the sEH improved insulin sensitivity and reduced apoptosis on pancreas islet cells [92]. Similarly, inhibition of sEH activity by t-AUCB prevented hyperglycemia in a streptozotocin-induced T1D model, prevented islet β cell damage and improved glucose homeostasis [93]. Consistent with previous findings, t-AUCB also maintained glucose homeostasis by increasing the muscle capillary blood volume and microvascular blood flow in db/db mice [94].

Type 2 diabetes (T2DM) is a metabolic disease characterized by insulin resistance, β-cell dysfunction, and increased hepatic glucose production [95,96]. A growing body of evidence indicated that CYP450-EETs-sEH pathway regulated insulin resistance and therefore prevented the development of T2DM. Mice with gene knockout of Cyp2c44 exhibited significant decreased insulin sensitivity in peripheral tissue [97]. CYP2J3 overexpression in db/db mice improved insulin resistance, decreased endoplasmic reticulum stress by activating the AMPK and insulin receptor (IR)-PI3K-AKT signaling pathway [98,99]. Similar effects were observed in diabetes rat models with CYP2J3 overexpression, and the protective effects were associated with increased level of nitric oxide synthase in endothelial cells [93]. CYP2J2 overexpression-derived EETs or treatment with exogenous EETs also improved insulin resistance, increased glucose uptake and restored glucose homeostasis by activation of PPARγ [100]. Consistent with the effects of CYP450 overexpression, a dual EET agonist/soluble epoxide hydrolase inhibitor to HO-2-null mice increased EET levels and consequently increased insulin sensitivity and decreased serum inflammatory cytokines TNF-α and MCP-1 [101]. Although adipose tissue is a primary site of inflammation in T2DM, the role of inflammation in other metabolic active tissues, such as the liver, remains substantial in the development of T2DM. CYP2J2 overexpression alleviated hepatic inflammation, including reduced production of proinflammatory cytokines and decreased the infiltration of macrophages in the liver, which attenuated metabolic dysfunction in diabetic mice [100]. Additionally, the upregulation of SGLT2 expression via inhibiting NF-κB signaling pathway in renal tubular epithelial cells mediated the attenuation of insulin resistance by 14,15-EET [102].

### 5.2. EETs and Diabetic Complication

Diabetic complications, such as diabetic cardiomyopathy, nephropathy, and retinopathy arise in both type 1 and type 2 diabetes. Diabetic cardiomyopathy is defined by the existence of abnormal myocardial structure and performance in the absence of other cardiac risk factors in individuals with diabetes mellitus [103]. Cardiac-specific overexpression of CYP2J2 significantly attenuated cardiac insulin resistance and glucose, and subsequently alleviated diabetic cardiomyopathy. The underlying mechanisms include activation of insulin receptor and AMPK signaling pathways, up-regulation of PPARγ, and inhibition of nuclear factor of activated T cells c3 (NFAT) signal by enhanced atrial natriuretic peptide (ANP) production [104]. Similarly, recent studies have shown that exogenous and endogenous EETs inhibited macrophages accumulation and prevented macrophages polarization from M1 to M2 by reducing the expression of EPAC in the metabolic environment, which substantially relieved the systemic and adipose tissue inflammation and insulin resistance [86]. Further studies showed that sEH blocker t-AUCB potently ameliorated inflammation, cardiac remodeling, and hypertrophy in a model of insulin resistance, which was related to decreased infiltration of CD45- and F4/80-positive cells, downregulated expression of p-AKT, sEH, NF-κB, NOX4, and reduced fibrosis related proteins [105]. Similarly, sEH inhibitor AUDA attenuated cardiac remodeling and dysfunction in DCM by increasing autophagy and reducing apoptosis via activating Nrf2 signaling pathway [106].

**Figure 4:**
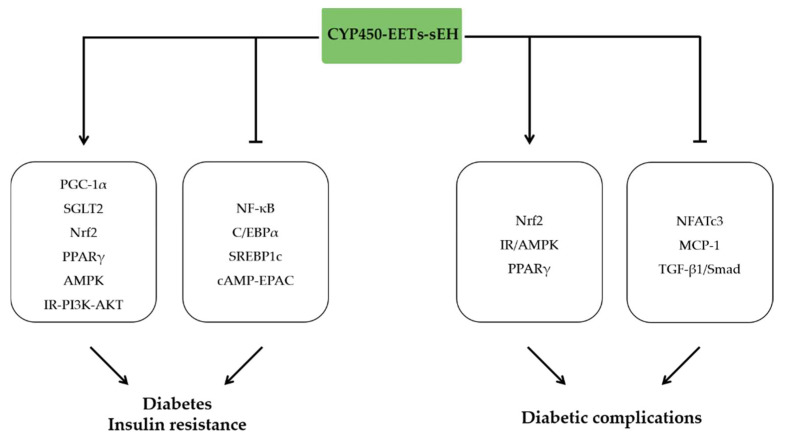
The anti-inflammatory effects of EETs in metabolic disorder. EETs ameliorate diabetes and insulin resistance by activating PGC-1α, PPARγ, AMPK, and IR-PI3K-AKT signaling pathways and inhibiting NF-κB, C/EBPα, and SREBP1c expression. EETs attenuate diabetic complications by increasing Nrf2, IR/AMPK, and PPARγ signaling pathways and inhibiting NFATc3 and TGF-β1/Smad signaling.

Diabetic nephropathy and retinopathy are severe complications leading to end-stage renal disease and blindness [107]. CYP2J2-specific overexpression in endothelial cells prevented renal damage induced by hyperglycemia and proteinuria, which was strongly related to the inhibition of TGF-β1/Smad signaling pathway [108]. Exogenous EETs treatment reduced the apoptosis induced by TNF-α and protected human proximal tubule cells from death [108], similar to the effects of CYP2J2 overexpression and EETs treatment. Pharmacological manipulation of sEH by t-AUCB in db/db mice significantly attenuated diabetic renal injury through reliving mitochondrial dysfunction and ER stress via inhibition of inflammation and autophagy [109]. A COX-2/sEH dual inhibitor PTUPB also significantly reduced urinary MCP-1 levels and renal cytokine expression, and therefore alleviated the development of diabetic nephropathy in rat [110]. In context of diabetic retinopathy, expression of CYP2B2 was significantly downregulated and 11,12-EET promoted angiogenesis in the retina under ischemia and hypoxia caused by diabetes [111,112] (Figure 4).

## 6. The Anti-Inflammatory Effects of EETs in Systemic Infectious Disease

**Figure 5:**
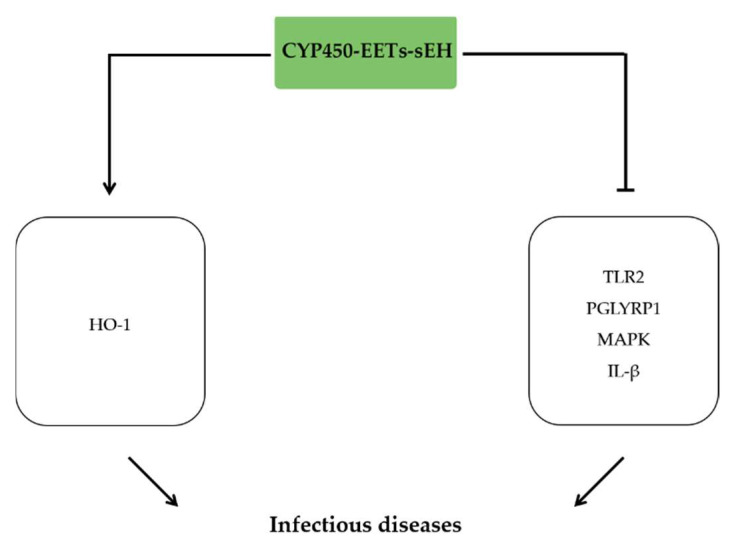
The anti-inflammatory effects of EETs in systemic infectious disease. EETs ameliorate infectious injury by activating HO-1 signaling pathway, inhibiting MAPK signaling pathway and reducing TLR2, PGLYRP1, and IL-1β expression.

Sepsis is a collection of syndromes and organ dysfunction induced by infection, which leads to imbalanced inflammatory response and immune homeostasis [113]. Evidence indicated that EETs inhibited macrophage activation and phagocytosis of *Streptococcus pneumoniae* by the downregulation of TLR2 and PGLYRP1 expression [114]. However, another study showed that EETs improved macrophage phagocytic ability to clear bacteria by suppressing mitogen-activated protein kinase signaling and decreasing pro-inflammatory cytokines release [115]. In LPS-induced RAW264.7 cells, exogenous 11,12-EET and 14,15-EET supplementation or TPPU treatment suppressed LDH release and inflammatory injury by inhibiting the expression of IL-1β and TNF-α [116]. These results suggested that EETs are potentially beneficial under systemic infectious conditions due to their pro-phagocytic ability and anti-inflammatory property. The in vitro studies confirmed that the sEH inhibition prolonged the median survival time and reduced the mortality in septic mice induced by LPS treatment and cecum ligation and puncture (CLP) surgery, respectively [115,117]. Consistent with the sEH inhibitor, COX-2/sEH dual inhibitor PTUPB inhibited inflammatory storm and oxidative stress by increasing HO-1 in the CLP model [118] (Figure 5).

## 7. The Anti-Inflammatory Effects of EETs in Respiratory Diseases

Acute lung injury is a common respiratory disease characterized by severe inflammation, which has infectious and non-infectious causes. Numerous studies have indicated that EETs derived from genetic or pharmacological manipulations of AA-CYP450-EETs-sEH pathway relieved lung injury in the context of different pathophysiological environments. CYP2J2 overexpression relieved pulmonary arterial hypertension by inhibiting the inflammatory response, oxidative stress, and apoptosis via activation of PPARγ and PI3K/AKT signaling pathway [119]. CYP2J2 also attenuated ischemia/reperfusion injury of the lung by decreasing oxidative stress and cell apoptosis via inhibiting NADPH oxidase activity and activating PI3K/Akt pathway [120]. Beyond the protective effects of CYP450 overexpression, gene knockout of sEH exhibits beneficial effects to lung injury. sEH knockout increased the levels of EETs and heme oxygenase-1 activity, which inhibited the production of ROS and activation of NLRP3 inflammasome, and subsequently attenuated hyperoxic acute lung injury (HALI) [121]. Similarly, sEH activated Nrf2/ Keap1 pathway and therefore reduced the pulmonary edema and inflammation in hyperoxia-induced ALI [122]. Gene knockout of sEH also attenuated Ang II induced lung inflammation, including reduced inflammatory cytokines levels in bronchoalveolar lavage fluid, the capillary protein leak, and lung histological alterations [123]. Similar to the effects of sEH knockout, sEH inhibitor TPPU suppressed TREM-1 expression and inflammation via inhibiting macrophage NF-kB activation in LPS-induced lung injury [124]. Meanwhile, the survival rate of LPS-induced acute lung injury (ALI) mice was improved by TPPU, which is associated with reduced neutrophil infiltration and decreased pro-inflammatory cytokine levels via EETs elevation [116].

Pulmonary toxicity and pulmonary fibrosis are the major adverse effects of anti-tumor therapy. In bleomycin-induced pulmonary injury mice model, AUDA significantly attenuated inflammation by inhibiting p38-MAPK signaling pathways, and therefore reduced inflammatory cell accumulation and decreased the expression of interleukin-1β, TGF-β, and matrix metalloproteinase 9 (MMP-9) [125]. Consistently, another sEH inhibitor TPPU decreased TGF-β1, IL-1β, and IL-6 levels in the serum of bleomycin-treated mice, and subsequently inhibited the inflammation and fibrosis of the lung [126].

In a tobacco smoke-exposed rat model, sEH inhibitor markedly reduced the number of neutrophils, macrophages, and lymphocytes in bronchoalveolar lavage [127]. Meanwhile, sEH inhibition or knockout alleviated the lung inflammation, emphysematous changes, and improved lung function by reducing IFN-γ and vascular remodeling-related growth factor [128,129]. In vitro experiments showed that cigarette smoke extract (CES) inhibited the expression of CYP2C8 and increased expression of sEH [130]. In turn, 11,12-EET or 14,15-EET treatment reduced the CES induced interleukin-8 production in bronchial epithelial cells by inhibiting the phosphorylation of p38 MAPK pathway [130]. Consistently, treatment with 14,15-EET reduced the release of inflammatory cytokine IL-6, IL-8, and MCP-1, accumulation of Nrf2 and expression of HO-1 in human bronchial epithelial cell [131].

**Figure 6:**
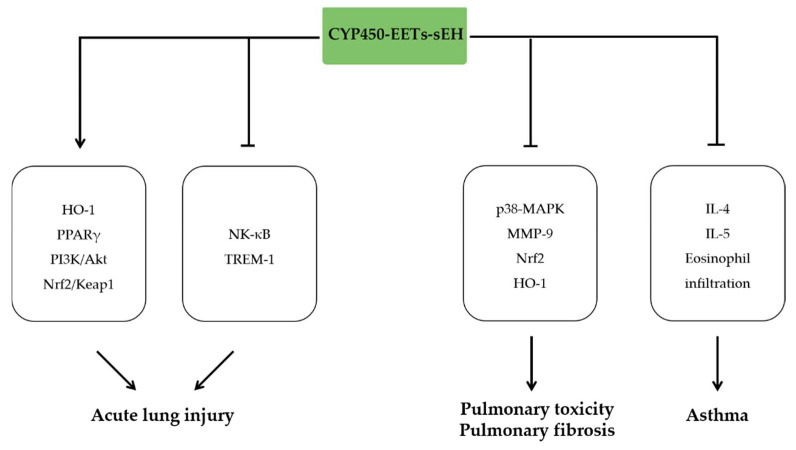
The anti-inflammatory effects of EETs in respiratory diseases. EETs ameliorate acute lung injury by activating HO-1, PPARγ, PI3K/AKT, and Nrf2/Keap1 signaling pathways and inhibiting NF-κB and TREM. EETs attenuate pulmonary toxicity and pulmonary fibrosis by inhibiting Nrf2, p38-MAPK, and HO-1 signaling pathways. EETs also alleviate asthma by inhibiting eosinophil infiltration and IL-4 and IL-5 expression.

Asthma is a chronic disease of the respiratory system, which is closely associated with airway inflammation and hyperresponsiveness. Beyond the well-established role of leukotrienes (LTs) in pathophysiological process in asthma, extensive evidence suggested that EETs derived from sEH inhibition prevent the development of asthma [132,133]. In ovalbumin (OVA)-induced asthma model, sEH inhibitor t-TUCB decreased the eosinophils and inflammatory cytokines in lavage of lung [134]. Similarly, t-TUCB reduced the cytokines (IL-4, IL-5) and chemokines (Eotaxin and RANTES) secretion by Th2 cells, and subsequently decreased recruitment of inflammatory cells infiltration into the lungs and airways [134]. In OVA-induced chronic mice model, AUDA reduced eosinophil infiltration into the lung tissue, and downregulated remodeling-related molecules [135]. In *A. alternate*-induced mouse model, t-TUCB significantly inhibited the development of structural changes in the allergic airways, reduced eosinophilic infiltration in lung tissue, and decreased allergen-induced IL-4, IL-13 levels [136] (Figure 6).

## 8. The Anti-Inflammatory Effects of EETs in Neurological Diseases

**Figure 7:**
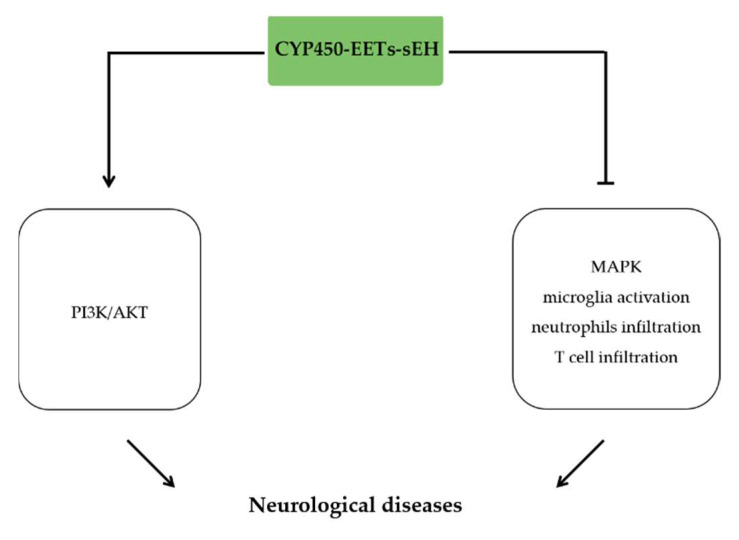
The anti-inflammatory effects of EETs in neurological diseases. EETs alleviate neurological injury by activating PI3K/AKT signaling pathway, reducing microglia activation, neutrophils and T cell infiltration, and inhibiting MAPK signaling pathway.

Neurological diseases are a series of multi-factorial diseases accompanied with proceed neuronal cell death and brain damage, in which neuroinflammation plays a key role [137]. Ischemic stroke results in neurological injury, accumulating evidence indicated that EETs derived from CYP450 overexpression or sEH inhibition protected brain against the ischemic injury by inhibiting inflammatory-related pathological process [138,139,140,141,142]. CYP2J2 overexpression decreased cerebral infarct size and increased brain perfusion by activation of the PI3K/AKT signaling pathway [138]. In the rat MCAO model, treatment with AUDA or 14,15-EET effectively promoted angiogenesis, reduced astrogliosis and neuronal apoptosis, as well as suppressed microglia activation and inflammatory response [140]. In spinal cord injury rat model, treatment with AUDA markedly reduced local inflammatory response, which is associated with reduced microglia activation and IL-1β expression, as well as the decreased infiltration of neutrophils and T lymphocytes [141]. In the context of central post-stroke pain (CPSP), administration of TPPU attenuated ER stress and MAPK signaling, and therefore reduced microglia and astrocytes activation in rat [142] (Figure 7).

## 9. The Anti-Inflammatory Effects of EETs in Liver Disease

Non-alcoholic fatty liver disease (NAFLD) and non-alcoholic steatohepatitis (NASH) is linked to liver inflammation as well as hepatocyte death [143]. A growing body of evidence indicated that NAFLD is accompanied with downregulated CYP450 expression, enhanced sEH activity and reduced EETs concentration [144], suggesting the crucial roles of AA-CYP450-EETs-sEH in pathophysiology of NAFLD. sEH inhibition or deletion significantly decreased liver inflammation, including attenuation of macrophage accumulation and reduction of inflammatory response and ER stress. These protective effects were associated with inhibition of JNK, p38 PERK, IRE1α, and ATF6 signaling pathway [145,146]. In line with the effects of sEH gene knockout, administration of EET analogues reduced hepatic fibrosis, insulin resistance, and inflammation induced by HFD, which is associated with an increase in HO-1-PGC1α and enhanced insulin receptor phosphorylation [147]. COX-2/sEH dual inhibitor PTUPB relived high-fat diet induced non-alcoholic fatty liver disease via inhibiting NLRP3 inflammasome activation in mice [148].

**Figure 8:**
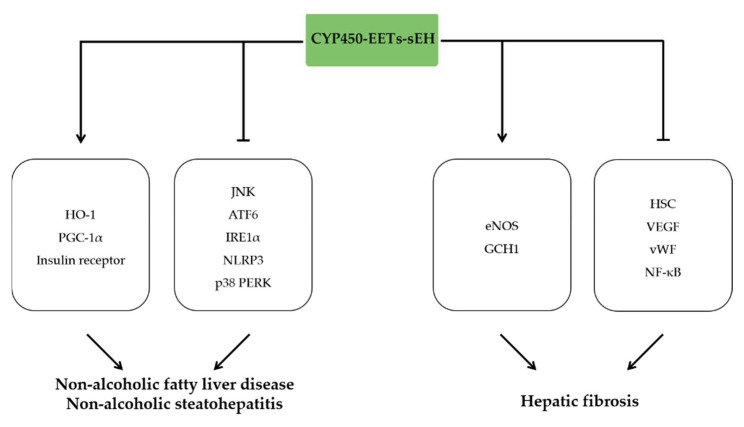
The anti-inflammatory effects of EETs in liver disease. EETs ameliorate non-alcoholic fatty liver disease and non-alcoholic steatohepatitis by activating HO-1, PGC-1α, and IR signaling pathways, reducing JNK, ATF6, and IRE1α expression and inhibiting NLRP3 and p38 PERK signaling pathways. EETs attenuate hepatic fibrosis by increasing eNOS and GCH1 and inhibiting HSC, VEDF, vWF, and NF-κB signaling pathways.

Hepatic fibrosis is a pathogenic character of several liver diseases that can be induced by pro-inflammatory cytokines. sEH gene deletion or sEH inhibition by TPPU or t-TUCB improved carbon tetrachloride (CCl4)-induced liver fibrosis in mice by decreasing expression and activity of matrix metalloprotease [149]. Meanwhile, sEH inhibition also inhibited hepatic fibrosis by attenuating inflammation factors, ER stress, and oxidative stress [149,150]. Portal Hypertension (PTH) is a complication of hepatic fibrosis, and there is currently no specific treatment for PTH. In CCL4-induced rat model, PTUPB administration reduced portal pressure, increased arterial wall thickness, prevented angiogenesis, and reduced vascular remodeling via regulating VEGF and von Willebrand factor (vWF) [151]. Additionally, PTUPB rescued the impaired sinusoidal vasorelaxation by upregulating endothelial eNOS and GTP-cyclohydrolase 1 (GCH1) [151]. Similarly, sEH inhibitor t-TUCB also lowered portal hypertension in cirrhotic rats by ameliorating endothelial dysfunction and liver fibrosis, which was primarily due to the inhibition of HSC activation and NF-κB signaling by EETs [152] (Figure 8).

## 10. The Anti-Inflammatory Effects of EETs in Kidney Injury

**Figure 9:**
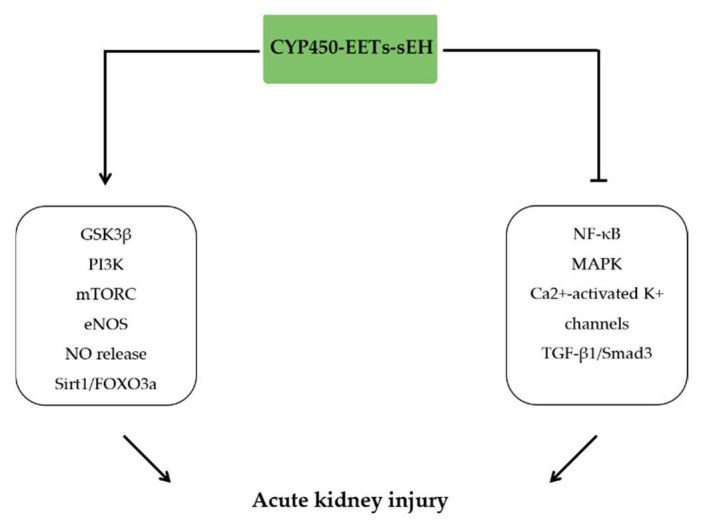
The anti-inflammatory effects of EETs in kidney injury. EETs ameliorate acute kidney injury by activating GSK3β, PI3K, mTORC, eNOS, and Sirt/FOXO3a signaling pathways and inhibiting NF-κB, MAPK, TGF-β1/Smad3, and Ca^2+^-activated K^+^ channels.

The CYP450-EETs-sEH system is important in preventing kidney damage caused by inflammation. EETs derived from CYP2J2 overexpression protected I/R-induced rat acute kidney injury (AKI) through activation of autophagy by upregulating the Sirt1/FOXO3a signaling pathway [153]. Previous findings have indicated that 14,15-EET increased the p-eNOS and NO release by blocking the Ca^2+^-activated K^+^ channels, which caused afferent arteries dilatation and reduced renal inflammation [154]. Intraperitoneal injection of 14,15-EET also decreased I/R-induced mice AKI via relieving renal tubules dilation by increasing GSK3β phosphorylation and restoring p-GSK3β/GSK3β ratio [155]. Consistent with previous findings, sEH depletion and sEH inhibitor treatment has been reported to attenuate the renal inflammation and injury. sEH depletion alleviated interstitial inflammation and renal fibrosis by inhibiting the TGF-β1/Smad3 and NF-κB signaling, decreasing infiltration of neutrophils and macrophages and preventing ROS induced cell death in rat unilateral ureteral obstruction model [156]. In LPS-induced kidney injury model, podocyte sEH-deficient and TPPU treatment relieved kidney injury via reducing NF-κB and MAPK activation and attenuating ER stress [157]. Administration of sEH inhibitor nb-AUDA and AUDA also attenuated the renal injury in cisplatin-induced mice AKI model [158]. Similar to the effects of sEH depletion or inhibition, the 14,15-EET analog alleviated the I/R-induced AKI by reducing tubular apoptosis and inflammatory cell infiltration through increasing PI3K and mTORC2-related phosphorylation of Akt [18]. 14,15-EET analogue also alleviated the cyclosporine-induced rat renal dysfunction by inhibition of inflammatory cells infiltration into the kidney and reduction of renal fibrosis [159] (Figure 9).

## 11. The Anti-Inflammatory Effects of EETs in Arthritis

Arthritis is the swelling and tenderness of one or more joints. The most common types of arthritis are osteoarthritis and rheumatoid arthritis. Administration of TPPU attenuated articular tissue injury, hyperalgesia, edema, and expression of pro-inflammatory factors by inhibiting the activation of Th1 and Th17 cells and enhancing Treg cells in collagen-induced arthritis [160]. In albumin-induced rat temporomandibular joint arthritis model, pretreatment with TPPU inhibited the hypernociception and leukocyte migration. These protective effects were associated with decreased inducible nitric oxide synthase (iNOS) expression, reduced pro-inflammatory cytokine and upregulated anti-inflammatory cytokine [161]. Similarly, sEH inhibitor reduced the hyperalgesia induced by inflammation in the early- and post-inflammatory phase in K/BxN mouse model of arthritis [162].

**Figure 10:**
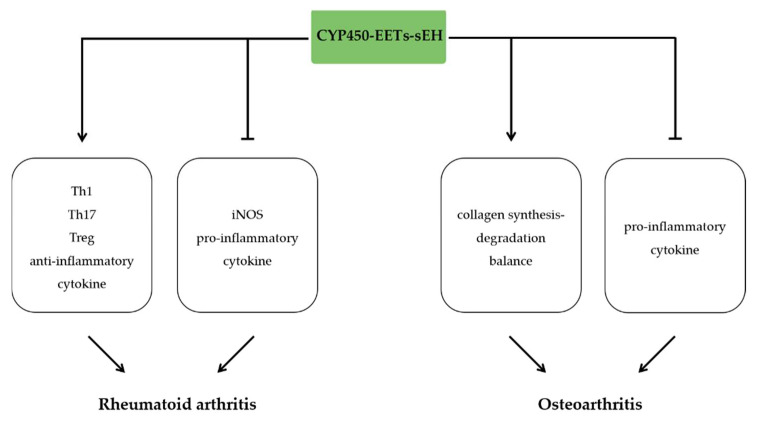
The anti-inflammatory effects of EETs in arthritis. EETs ameliorate rheumatoid arthritis by increasing Th1, Th17, Treg, and anti-inflammatory cytokines and reducing iNOS and pro-inflammatory cytokines. EETs ameliorate osteoarthritis by promoting collagen synthesis-degradation balance and reducing pro-inflammatory cytokines.

Osteoarthritis causes pain and bone deterioration induced by increased prostaglandins (PGs) and inflammatory cytokines [163]. A recent study has indicated that SNPs of the soluble EH gene EPHX2 and EET/DHET pathway are associated with knee pain caused by OA [164]. Interestingly, in destabilization of the medial meniscus (DMM) model of OA pain, treating of TPPU markedly reduced plasma levels of 8,9-DHET and 14,15-DHET without altering EETs concentrations [164]. Meanwhile, t-TUCB alleviated inflammatory pain in canine osteoarthritis, which is associated with decreased inflammatory cytokines, including IL-6, TNF-α, and reduced cytotoxicity by elevation of EETs [163]. Similarly, t-TUCB coupled with COX inhibitor reduced joint pain, prostanoid responses, and restored collagen synthesis-degradation balance in LPS-induced horse model [165] (Figure 10).

## 12. Conclusions and Future Direction

**Figure 11:**
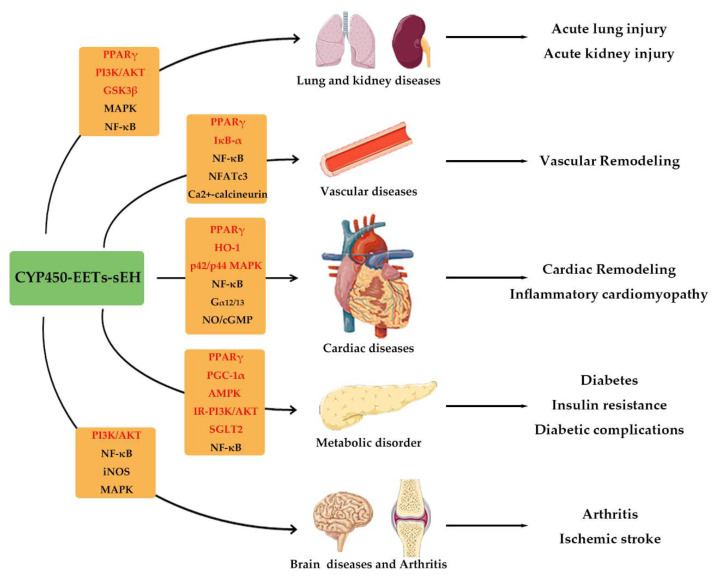
The major signaling pathways and beneficial effects of CYP450-EETs-sEH system in inflammatory-related diseases. EETs attenuate vascular inflammation and remodeling by regulating PPARγ, NF-κB/IκB-α, NFATc3, mitoK_ATP_, and Ca^2+^-calcineurin. EETs also process direct and indirect anti-inflammatory properties in the myocardium, and therefore alleviate inflammatory cardiomyopathy and cardiac remodeling. The related molecular mechanisms including PPARγ, NF-κB, HO-1, Gα12/13, NO/cGMP, and p42/p44 MAPK. Moreover, EETs play substantial roles in relieving inflammation under other pathophysiological environments, such as diabetes, sepsis, lung injuries, neurological disease, hepatic diseases, kidney injury, and arthritis. The related molecular mechanisms including PPARγ, NF-κB, PI3K/AKT, GSK3β, AMPK, MAPK, et al. For the whole figure, the red represents activation, and the black represents inhibition in the orange textbox.

Rapid progress has been made in evaluating the roles of AA-CYP epoxygenase-EETs-sEH pathway in the pathophysiology of human diseases, of which anti-inflammatory actions have gained great recent interest. EETs exhibit potent protective effects on the prevalence of inflammation-related diseases, including cardiovascular remodeling, metabolic disorders, sepsis, lung injuries, neurodegenerative disease, hepatic diseases, kidney injury, and arthritis (Figure 11). Moreover, the EETs analog and sEH inhibitors also show the strong anti-inflammatory properties in many systematic illnesses, such as heart failure, diabetes, neuro-inflammatory pains, suggesting that pharmacological manipulations of AA-CYP450-EETs-sEH systems are of great potential in clinical applications.

Although, great progress has been made in this field, there remain many important questions to be answered. (1) The global metabolic changes of AA under different pathological conditions, such as cardiac remodeling and metabolic disorders, should be further investigated by metabolomics. (2) The global effects of CYP epoxygenases and their metabolites, EETs, on inflammatory diseases remain to be clarified by genomics and proteomics. (3) Whether the adverse effects of EETs, such as promoting metastasis, during the course of therapy will occur remains unknown. (4) The receptors of CYP epoxygenase metabolites, such as EET receptor, remain to be identified. Importantly, the identification and characterization of these receptors and development of receptor-specific agonists and antagonists could have great therapeutic potential.

## Abbreviations

AA	arachidonic acid
EET	epoxyeicosatrienoic acid
sEH	soluble epoxy hydrolase
PLA2	phospholipase A2
COX	cyclooxygenase
LOX	lipoxygenase
5-LOX	5-lipoxygenase
ALOX5	arachidonate 5 lipoxygenase
CYP	cytochrome P450
HETEs	hydroxyeicosatetraenoic acids
DHETs	dihydroxyeicosatrienoic acid
ICM	inflammatory cardiomyopathy
LPS	lipopolysaccharide
CHD	coronary heart disease
NSAIDs	non-steroidal anti-inflammatory drugs
ROS	reactive oxygen species
AAVs	adeno-associated virus
Ang II	angiotensin Ⅱ
NF-κB	nuclear factor-κB
CAM	cellular adhesion molecule
VCAM-1	vascular cell adhesion molecule-1
ICAM-1	intercellular adhesion molecule-1
IKK	IκB kinase
IκB-α	inhibitor κB-α
HSVEC	human saphenous vein endothelial cells
PA	primary aldosteronism
AAC	abdominal aortic calcification
AAA	abdominal aortic aneurysm
PGC-1α	peroxisome proliferation-activated receptor γ coactivator α
T1DM	Type 1 diabetes
T2DM	Type 2 diabetes
SGLT2	sodium-dependent glucose transporters 2
AMPK	AMP-activated protein kinase
ANP	atrial natriuretic peptide
NF-AT	nuclear factor of activated T cells
ALI	acute lung injury
HALI	hyperoxic acute lung injury
MMP-9	matrix metalloproteinase 9
CES	cigarette smoke extract
OVA	ovalbumin
MAPK	mitogen-activated protein kinases
IR	insulin receptor
AD	Alzheimer’s disease
PD	Parkinson’s disease
TH	tyrosine hydroxylase
CPSP	central post-stroke pain
NAFLD	Non-alcoholic fatty liver disease
NASH	Non-alcoholic steatohepatitis
vWF	von Willebrand factor
PTH	portal hypertension
CCl4	carbon tetrachloride
eNOS	endothelial nitric oxide synthase
iNOS	inducible nitric oxide synthase
GCH1	GTP-cyclohydrolase 1
GSK3β	glycogen synthase kinase 3β
AKI	acute kidney injury
RA	rheumatoid arthritis
OA	osteoarthritis
PGs	prostaglandins
DMM	destabilization of the medial meniscus
MPTP	1-methyl-4-phenyl-1,2,3,6-tetrahydropyridine
sEHIs	soluble epoxy hydrolase inhibitors
AUDA	12-(3-adamantan-1-yl-ureido) dodecanoic acid
APAU	1-(1-Acetypiperidin-4-yl)-3-adamantanylurea
TPPU	1-(1-propanoylpiperidin-4-yl)-3-[4-(trifluoromethoxy)phenyl]urea
PTUPB	4-(5-phenyl-3-(3-[3-(4-trifluoromethyl-phenyl)-ureido]-propyl)S-pyrazol-1-yl) benzenesulfonamide
nb-AUDA	n-butyl ester of 12-(3-adamantan-1-yl-ureiido)-dodecanoic acid
t-AUCB	trans-4-[4-(3-Adamantan-1-yl-ureido)-cyclohexyloxy]-benzoic acid
t-TUCB	trans-4-[4-[3-(4-Trifluoromethoxy-phenyl)-ureido]-cyclohexyloxy]-benzoic acid
